# Swine Group A rotavirus vaccines: current status, adjuvant strategies, challenges, and future perspectives

**DOI:** 10.3389/fvets.2026.1896748

**Published:** 2026-07-17

**Authors:** Zhang Zhen, Ma Baihe, Zhou Hongmiao, Guo Meiliang, Liu Shuhua, Li Lianrui

**Affiliations:** 1 College of Animal Science and Technology, Tarim University, Alar, Xinjiang, China; 2 Tarim Animal Husbandry Science and Technology Key Laboratory of Xinjiang Production and Construction Corps, Alar, Xinjiang, China; 3 Tarim Animal Disease Diagnosis and Prevention Engineering Laboratory of Xinjiang Production and Construction Corps, Alar, Xinjiang, China; 4 Bayinguoleng Vocational and Technical College, Korla, Xinjiang, China

**Keywords:** adjuvant, porcine Group A rotavirus, vaccine, vaccine formulation, viral diarrhea

## Abstract

Swine Group A rotavirus is one of the primary intestinal pathogens causing severe diarrhea in newborn piglets, with high incidence and mortality resulting in substantial economic losses to the global swine industry. Vaccination, alongside rigorous biosecurity measures, remains the core strategy for controlling porcine Group A rotavirus infection; however, continuous genetic variation via genomic reassortment, point mutations, and diverse G/P genotype combinations drives the constant emergence of new circulating strains that may evade immune protection from existing vaccines. These genetic changes may also give rise to highly pathogenic variants, increasing large-scale outbreak risk. Therefore, developing vaccines that provide stable, broad cross-protection against viral strain diversity is particularly critical. Vaccine adjuvants play a vital role in enhancing and modulating immune responses, broadening protection spectra, and reducing the antigen dose required for protection especially valuable when facing rapidly changing genotypes or limited vaccine antigen supplies. In recent years, advances in adjuvant design and formulation strategies have clearly improved the immunogenicity and duration of protection of swine Group A rotavirus vaccines, as evidenced by significantly reduced diarrhea incidence and viral shedding intensity in vaccinated piglets. Building on an overview of the current status, advantages, and challenges of different porcine Group A rotavirus vaccine types, this review focuses on the mechanisms of action, application advantages, existing limitations, and methodological strategies of adjuvants. It further analyzes the potential contribution of adjuvant strategies to developing a universal porcine rotavirus vaccine capable of broad, long-lasting cross-protection. Finally, this review identifies key unresolved scientific questions and critical knowledge gaps in the field, with the aim of guiding future research priorities.

## 1 Introduction

Porcine Group A rotavirus (RVA) is a highly contagious intestinal pathogen that primarily infects pigs, with newborn piglets being particularly susceptible. The virus belongs to the genus Rotavirus within the family Reoviridae, and its genome consists of 11 segments of double-stranded RNA (1, 2). Based on antigenic differences in the viral capsid proteins VP7 and VP4, the virus can be classified into G and P types. To date, multiple G/P genotype combinations have been identified in pig populations, such as G3, G4, G5, G9, and G11 paired with P[6], P[7], and P[13], among others (3, 4). The segmented genome of porcine Group A rotavirus allows it to exchange RNA segments through genetic reassortment during co-infections, producing progeny viruses with new antigenic characteristics (5). These reassortment events, akin to “antigenic shift,” act in concert with continuous antigenic drift caused by the accumulation of point mutations, leading to the constant diversification of field-circulating strains. This results in low cross-immunity against new strains within pig populations, potentially triggering severe outbreaks on pig farms (1, 6, 7). Furthermore, the persistent circulation and rapid evolution of porcine rotavirus within pig populations further exacerbate the risk of its transmission across different regions and farms, posing a long-term challenge that requires integrated biosecurity protocols and systematic vaccination for effective prevention and control (7).

Infection with porcine Group A rotavirus primarily causes watery diarrhea, dehydration, and vomiting in piglets aged 1–8 weeks; the incidence rate can reach 80–100%, with a mortality rate ranging from 10 to 50%. If secondary bacterial infections occur, the losses are even more severe (1, 8). In addition to directly causing piglet mortality, the infection also leads to growth retardation and reduced feed conversion efficiency, imposing a significant economic burden on the global swine industry. It is estimated that the U.S. swine industry incurs annual economic losses of millions of USD due to rotavirus-associated production losses, while losses in developing regions are generally higher due to variable biosecurity and husbandry standards (1, 3, 9). These estimates are derived from farm-level surveillance and production economic models, with inherent uncertainty due to co-infections and underreporting in low-resource settings.

In combination with farm-level biosecurity practices, vaccination remains one of the most effective means of preventing infection with porcine Group A rotavirus and limiting its transmission. However, current vaccine development and deployment face three core challenges. First, current commercial vaccines are mostly designed based on locally dominant G/P genotype strains, but the high frequency of genetic reassortment and point mutation causes continuous shifts in circulating strain spectra, leading to declining antigenic matching and weakened protective efficacy (1, 10, 11). Second, vaccine-induced immune responses are highly genotype-specific, providing insufficient cross-protection against heterologous strains and leaving herds vulnerable to emerging variants (12). Third, the immaturity of the neonatal piglet immune system and maternal antibody interference further limit the realization of vaccine efficacy, especially for mucosal and cellular immune responses (13, 14).

To overcome this limitation, the development of next-generation porcine rotavirus vaccines containing novel adjuvants can significantly enhance the intensity and durability of the immune response (15, 16). Adjuvants play a key role in enhancing vaccine efficacy by activating innate immune pathways, enhancing antigen presentation, and promoting long-lasting adaptive immunity (13). In the context of Group A porcine rotavirus vaccines, factors such as antigenic drift, strain diversity, the immaturity of the newborn piglet’s immune system, and maternal antibody interference often limit the protective efficacy of vaccines, making the addition of adjuvants particularly important (13, 14). Currently, various adjuvant strategies, such as Toll-like receptor agonists and nanoparticle carriers, have been shown to induce cross-protective immunity against multiple G/P genotype strains, demonstrating promising application prospects (17, 18).

The core objective of this review is to provide a fundamental overview of porcine Group A rotavirus and to systematically summarize the types, mechanisms of action, and inherent limitations of traditional porcine rotavirus vaccines, particularly the insufficient cross-protection resulting from homotypic protection. Building on this foundation, the review focuses on the key role of adjuvants in enhancing the immunogenicity and overall efficacy of existing vaccines, as well as their ability to induce broad cross-protective responses. We further define the evidence gaps between enhanced immunogenicity and confirmed cross-protection for adjuvant strategies, and critically evaluate emerging universal vaccine approaches. Finally, we outline key unresolved questions and future research directions to advance the development of next-generation broadly protective vaccines.

## 2 Methods

This review employs a narrative review approach, aiming to comprehensively synthesize current knowledge regarding the pathogenesis of porcine Group A rotavirus, existing vaccine types and their mechanisms of action, current porcine rotavirus vaccination strategies (including inactivated vaccines, live attenuated vaccines, and subunit vaccines currently under development), and the impact of adjuvant strategies on vaccine efficacy. It also examines the molecular epidemiology of porcine rotavirus, immunoprotective effects, and related prevention and control strategies. A narrative review was chosen to facilitate a broad discussion from multiple perspectives including virology, immunology, vaccine development, and veterinary vaccine regulation rather than conducting a quantitative meta-analysis.

Relevant literature was obtained by searching multiple databases and official sources, including Google Scholar, PubMed, the World Organisation for Animal Health (WOAH), the Food and Agriculture Organization of the United Nations (FAO), the United States Department of Agriculture (USDA), and the official websites of China’s Ministry of Agriculture and Rural Affairs. The literature search was conducted up to 2026 with no restrictions on publication date. Included literature comprised peer-reviewed scientific studies, surveillance reports, and guidance documents published by official agencies and industry organizations. This approach allows for the integration of recent emerging trends while preserving historical findings and foundational data regarding swine rotavirus vaccine development. No language restrictions were imposed; however, only literature with full-text availability in English or Chinese was included. Inclusion criteria encompassed research papers, official reports, and technical guidelines regarding the composition, efficacy, cross-protection effects, adjuvant strategies, immunization regimens, molecular epidemiology of circulating strains, and prevention and control recommendations for swine Group A rotavirus vaccines. Exclusion criteria included non-peer-reviewed opinion pieces, unpublished data, and studies not directly related to swine group A rotavirus vaccines. As a narrative review, this study has inherent limitations: it did not employ systematic quantitative synthesis or meta-analysis methods, and there may be some selection bias; the results and conclusions depend on the availability and quality of published information and official reports within the search timeframe. Nevertheless, by synthesizing information from multiple authoritative databases and official sources, this review provides a relatively comprehensive overview of recent advances in swine Group A rotavirus vaccines and their adjuvant strategies.

## 3 Epidemiology, genetic diversity, and potential for cross-species transmission of porcine Group A rotavirus

Porcine Group A rotavirus is a major pathogen threatening the global swine industry, causing persistent harm to the health of newborn piglets through endemic circulation and sporadic large-scale outbreaks. Although porcine Group A rotavirus has not caused a global pandemic like human influenza viruses, specific G/P genotype combinations resulting from genetic reassortment have rapidly spread through pig populations in many regions of the world and caused severe clinical outbreaks. For example, from the late 1990s to the early 2000s, genotype combinations such as G9P[7] and G9P[13] were successively detected in pig populations across Asia, Europe, and the Americas; the G5 genotype has long dominated in parts of South America; and rare genotypes such as G11 and G26 have emerged in multiple countries in recent years, suggesting that the viral antigenic spectrum is continuously expanding (3, 19–21). These antigenic shifts, triggered by genomic segment reassortment, combined with the gradual accumulation of point mutations in the VP7 and VP4 capsid proteins (antigenic drift), have led to the continuous diversification of field-circulating strains. Consequently, traditional vaccines prepared using a limited number of strains sometimes fail to fully match the circulating strains (12, 22).

Porcine Group A rotavirus possesses an 11-segment double-stranded RNA genome, in which the genes encoding VP7 and VP4 determine the G-type and P-type, respectively. To date, at least 12 G-types and 15 P-types have been identified in pig populations, resulting in an extremely complex combination of the two (23). The virus’s own RNA-dependent RNA polymerase lacks proofreading functions, introducing high-frequency mutations during replication. It is estimated that the nucleotide substitution rate per site in the rotavirus genome is approximately 1.0 × 10^−3^ to 2.0 × 10^−3^ per year, driving the continuous emergence of new antigenic variants (24–27). This high genetic heterogeneity enables the virus to evade both the natural immunity of the sow herd and the neutralizing antibody response induced by vaccines. Even in large-scale pig farms with high vaccination coverage, diarrhea outbreaks caused by new variants still occur frequently, resulting in significant morbidity and mortality (1, 28). According to data from the World Organisation for Animal Health (OIE) and multi-country surveillance, the herd prevalence of rotavirus diarrhea in newborn piglets can reach 50–80%, and in farms lacking effective prevention and control measures, the mortality rate can reach 10–50% (3, 29, 30). It is estimated that in Asia alone, the direct and indirect economic losses caused by porcine Group A rotavirus infection amount to hundreds of millions of RMB annually, and the swine industries in Europe and the United States have similarly suffered severe economic impacts (1, 31). Although outbreaks of other intestinal pathogens, such as porcine epidemic diarrhea virus, have diverted attention to some extent in recent years, porcine group A rotavirus remains one of the most common viral pathogens causing diarrhea in suckling piglets (1, 2).

Porcine Group A rotavirus has a clear potential for cross-species transmission. Swine populations not only harbor their own unique rotavirus genotypes but can also be infected by human rotaviruses; in particular, genotypes common in human populations worldwide, such as G1, G2, and G4, have been repeatedly detected in swine populations (32–35). Pigs are regarded as important “mixing vessels” for rotavirus genetic reassortment. Their intestinal epithelial cells simultaneously express functional receptors for both human and porcine rotaviruses, making pigs susceptible to infection by both porcine and human rotaviruses (36). When both viruses co-infect the same host cell, gene segment reassortment occurs very easily, resulting in progeny viruses with new antigenic combinations. This reassortment mechanism has led to the emergence of multiple human-swine reassortant rotavirus strains; for example, G1P(8) or G9P(8) reassortant strains reported in Brazil, India, and Cameroon, which carry a porcine genetic backbone and are capable of infecting humans and causing clinical diarrhea, highlighting the potential role of pig populations in interspecies transmission of rotaviruses and the emergence of new strains (32, 37–39). In 2023, Jampanil et al. found in a surveillance study of diarrhea-affected piglets in northern Thailand from 2016 to 2023 that the PoRVA infection rate surged from 11.6% in 2019 to 39.6% in 2023. They identified various genotype combinations, including G5P(23), G4P(23), G9P(23), among others. Phylogenetic analysis indicated that most strains carried a human-derived Wa-like gene backbone, suggesting that frequent human-to-pig genetic reassortment drives the emergence of new genotypes; the continuous evolution of reassortant viruses places suckling piglets at high risk of disease, further confirming the real-world risk of viral reassortment leading to new circulating strains (40). Although reports of human infection with porcine rotavirus remain sporadic and no sustained human-to-human transmission has been detected, seroepidemiological surveys indicate a high seroprevalence of anti-porcine rotavirus antibodies among exposed populations, such as pig farm workers, suggesting the possible existence of previously unrecognized asymptomatic or mild infections (41). While confirmed cases of human infection with porcine rotavirus remain sporadic, and no sustained human-to-human transmission has been documented to date, seroepidemiological data suggest underrecognized occupational exposure risk (41). The ongoing generation of human-swine reassortant strains through pig “mixing vessel” dynamics warrants continued epidemiological surveillance. This potential zoonotic risk further underscores the public health value of developing broad-spectrum vaccines to reduce viral circulation in pig populations and mitigate cross-species transmission opportunities (32, 39).

Preliminary estimates based on regional surveillance data indicate that direct and indirect economic losses from porcine Group A rotavirus infection reach hundreds of millions of RMB annually in Asia, with comparable production burdens reported in European and North American swine industries (1, 31). These figures should be interpreted with caution, as they are often extrapolated from partial farm data and do not fully account for confounding factors such as co-infections with other enteric pathogens and variable farm management levels.

## 4 Animal models for the evaluation of swine Group A rotavirus vaccines

Selecting an appropriate animal model is crucial for evaluating the efficacy of swine Group A rotavirus vaccines. An ideal animal model should be susceptible to porcine Group A rotavirus and capable of exhibiting intestinal pathological changes and clinical symptoms consistent with natural infection. Animal models currently used in porcine rotavirus vaccine research include suckling mice, newborn piglets, germ-free pigs, and newborn calves (42–45). The suckling rat model is simple to handle and relatively low-cost, and is often used for preliminary screening of vaccine immunogenicity and challenge protection experiments; however, since most field strains of porcine rotavirus cannot efficiently infect suckling rats, they often require serial passage for adaptation, and the course of diarrhea and intestinal lesions observed in suckling rats still differ somewhat from those in newborn piglets (46, 47). Newborn calves are similarly susceptible to Group A rotaviruses, and some porcine rotavirus strains can cause diarrhea in calves, making them suitable for evaluating the cross-protective efficacy of vaccines; however, their large size, high cost, and the fact that they are not a species-specific model for pigs limit their widespread application (48–50). Germ-free pig models can effectively eliminate interference from maternal antibodies and commensal microorganisms on the vaccine response and are of unique value in studying active immune responses and immune mechanisms; however, they cannot fully simulate the maternal antibody environment and complex microbial ecology found under field conditions (42, 48).

Among all the models described above, newborn piglets are considered the ideal evaluation model that most closely resembles clinical practice, as they are the natural hosts of porcine Group A rotavirus. Piglet intestinal epithelial cells express a range of rotavirus receptors and attachment factors, such as various histocompatibility group antigens (HBGAs), which closely resemble the receptor distribution in the intestines of human infants. This allows piglets to be directly infected by field strains of porcine rotavirus of different G/P genotypes without the need for prior adaptation (51, 52). Following infection, piglets exhibit watery diarrhea, dehydration, weight loss, and typical pathological changes such as small intestinal villous atrophy and intestinal epithelial vacuolization, which are highly consistent with the clinical manifestations and intestinal damage observed in naturally infected piglets (53). Regarding the immune response, following infection or vaccination, piglets produce rotavirus-specific intestinal secretory IgA, serum IgG, and neutralizing antibodies, and can induce virus-specific T-cell responses; their mucosal and systemic immune response characteristics are similar to those of human rotavirus infection (54). More importantly, the newborn piglet model can be used to simulate the field immunization protocol of “sow immunization–colostrum transfer–passive protection of piglets.” By immunizing pregnant sows and then challenging their suckling piglets with the virus, the model allows for a direct evaluation of the actual protective efficacy of maternal antibodies in piglets a capability that other small animal models cannot easily replicate (55).

In addition to evaluating vaccine efficacy, piglet models have demonstrated unique advantages in research on mucosal vaccine delivery technologies and novel adjuvant strategies. The intestinal mucosal barrier in piglets, along with the distribution and function of associated lymphoid tissues, provides a reliable platform for systematically evaluating the ability of vaccines to induce intestinal mucosal immunity via oral or intranasal routes (1, 51, 52). Early exploratory studies involved oral immunization of sows with liposome-encapsulated inactivated porcine rotavirus antigens combined with intestinal adjuvants. These studies observed a significant increase in specific IgA and neutralizing antibody titers in colostrum, confirming the potential of lipid carriers to enhance the efficacy of mucosal vaccines (53, 54). Building on this, researchers further developed polymeric nanoparticle carriers, such as polylactic-co-glycolic acid (PLGA) encapsulating VP4 or VP6 protein antigens for intramuscular or intranasal immunization, which induced stronger intestinal sIgA responses and systemic antibody responses in newborn piglets. These vaccines also demonstrated partial cross-protection against challenge with heterologous strains and showed a trend toward reduced viral shedding and shorter duration of diarrhea, even when mucosal antibody levels were relatively modest (55–57). In recent years, nanodelivery systems based on biodegradable materials such as chitosan have been used to encapsulate inactivated porcine Group A rotavirus or recombinant subunit antigens. Following intranasal or oral immunization of piglets, these formulations not only induced high levels of rotavirus-specific intestinal sIgA and serum IgG but also enhanced the expression of cytokines such as IFN-*γ* and IL-17 in mesenteric lymph nodes, resulting in significantly reduced intestinal pathological damage and viral shedding following challenge (55, 58–60). In addition, strategies combining nanoparticles with adjuvants such as Toll-like receptor agonists or stimulators of interferon-like genes (STING) have also been evaluated in piglet models. For example, intranasal immunization of piglets with plant-derived cationic *α*-glucan nanoparticles combined with the double-stranded RNA analog poly(I:C) significantly enhanced cross-reactive sIgA in the nasal and intestinal mucosa as well as virus-specific T-cell responses in peripheral blood, and to some extent reduced intestinal viral load following challenge with a heterologous strain (61). Similarly, lipid nanoparticle-encapsulated mRNA or DNA vaccine platforms have also been explored in the field of porcine rotavirus vaccines in recent years; preliminary results indicate that these platforms can rapidly induce neutralizing antibodies and reduce viral shedding rates and diarrhea scores in piglets following challenge (45, 60). Overall, these studies using piglets as models clearly outline the evolutionary path of mucosal vaccines and adjuvant technologies for porcine Group A rotavirus: liposomes established the feasibility of mucosal delivery, polymeric nanoparticles enhanced immunogenicity and cross-protection potential, while emerging platforms such as plant-derived nanoparticles and lipid nanoparticles balance biocompatibility, scalable production, and cost-effectiveness. These studies further highlight the central role of the newborn piglet model in advancing novel swine Group A rotavirus vaccine and adjuvant strategies from the laboratory to clinical application.

## 5 Approved and commercially available swine Group A rotavirus vaccines

Globally, several vaccines targeting swine Group A rotavirus have been approved for marketing or have entered the commercialization phase, with significant differences in production processes and immunological characteristics across different platforms. Table 1 lists some swine Group A rotavirus vaccines that have been approved by major veterinary drug regulatory authorities (such as the U.S. Department of Agriculture (USDA) and the Chinese Ministry of Agriculture and Rural Affairs). The strain composition of swine Group A rotavirus vaccines is generally based on molecular epidemiological surveillance data regarding the distribution and variation of G and P types in endemic regions. These vaccines are typically formulated as multivalent vaccines to provide the broadest possible coverage against the dominant G/P genotype combinations circulating in local swine populations (10, 62, 63). The selection of vaccine strains often requires advance judgment based on surveillance data; if antigenic variation resulting from reassortment or point mutations occurs in the field and new dominant strains emerge, the actual protective efficacy of the vaccine may decline significantly (10, 64). Currently, commonly used swine Group A rotavirus vaccines can be broadly classified into three categories: live attenuated vaccines, inactivated vaccines, and recombinant subunit vaccines currently in development or early stages of application. These vaccines may be formulated as multivalent, bivalent, or monovalent (10, 48, 56, 65).

**Table 1:** Selected porcine Group A rotavirus vaccines licensed by major veterinary regulatory authorities.

Trade name	Vaccine type	Rotavirus strain (G/P type)	Other antigen components	Adjuvant	Manufacturer	Regulatory authority
PROSYSTEM® RCE	Inactivated	G5 (A1), G4 (A2)	*Clostridium perfringens* type C, *E. coli*	Mineral oil emulsion	Merck Animal Health (USA)	USDA
LitterGuard® LT-C	Inactivated	G5, G4	*Clostridium perfringens* type C, *E. coli*	Aluminum adjuvant	Zoetis (USA)	USDA
Trivalent Live Vaccine against TGEV, PEDV and Porcine Rotavirus (G5)	Live attenuated	G5	TGEV, PEDV	None (live vaccine)	Harbin Veterinary Research Institute, CAAS	Ministry of Agriculture and Rural Affairs, China
Bivalent Inactivated Porcine Rotavirus Vaccine (G5 + G9)	Inactivated	G5, G9	None	Oil emulsion (Montanide ISA 206)	Huapai Bioengineering Group	Ministry of Agriculture and Rural Affairs, China
Trivalent Inactivated Vaccine against TGEV, PEDV and Porcine Rotavirus (G9)	Inactivated	G9	TGEV, PEDV	Aluminum adjuvant + saponin	Luoyang Putai Biotechnology Co., Ltd.	Ministry of Agriculture and Rural Affairs, China

Information for US-licensed products is sourced from the U.S. Department of Agriculture (USDA), Animal and Plant Health Inspection Service (APHIS), Center for Veterinary Biologics (CVB), and product labels; data as of May 30, 2026 (see https://www.aphis.usda.gov/veterinary-biologics/licensed-product). Information for China-licensed products is sourced from the China Institute of Veterinary Drug Control (CIVDC) and product labels; data as of May 30, 2026 (see http://www.ivdc.org.cn).

### 5.1 Live attenuated vaccines

Live attenuated vaccines reduce the virulence of the pathogen while retaining its limited replicative capacity and immunostimulatory activity, enabling them to induce a protective immune response without causing clinical disease. In the development of swine Group A rotavirus vaccines, common strategies for obtaining attenuated strains include: serial passage in non-porcine cells or heterologous hosts to accumulate adaptive mutations, and the use of recombination technology to combine the protective capsid protein-encoding gene of a weak-virulence backbone strain with the corresponding gene from a circulating strain (10, 48, 66, 67). For example, a commercially available live vaccine is a trivalent live vaccine produced by combining an attenuated porcine rotavirus strain with attenuated strains of porcine transmissible gastroenteritis virus and porcine epidemic diarrhea virus. It is administered orally or intramuscularly to sows, and protective antibodies are transferred to piglets via the sows’ colostrum and milk (68). Oral administration mimics the natural fecal-oral route of infection for Group A porcine rotaviruses, allowing the vaccine virus to replicate to a limited extent in the intestinal epithelium, thereby inducing secretory IgA (sIgA) in the intestinal mucosa and a systemic immune response, thus establishing an immune barrier at the primary site of viral invasion (10, 42, 69, 70).

Although live attenuated vaccines are clinically recognized for their advantages of being non-injectable and inducing mucosal immunity, their cross-protection performance in the prevention and control of porcine rotavirus remains influenced by the degree of genotypic match between the vaccine strain and field-circulating strains. When new G/P genotype combinations resulting from reassortment emerge in pig herds, the heterologous immunity induced by the original live vaccine strain may be insufficient, leading to diarrhea outbreaks in individual farms (1, 10, 28, 71). Additionally, live attenuated vaccines are relatively sensitive to storage and transport temperatures, requiring strict maintenance of cold chain conditions during actual use on pig farms. Regarding safety, currently approved live attenuated porcine rotavirus vaccines generally exhibit good safety profiles; post-vaccination reactions such as transient loss of appetite or transient diarrhea are mostly mild. However, the use of live vaccines is generally not recommended for sows or piglets exhibiting significant immunosuppression (10). To date, there is insufficient evidence to suggest that registered live attenuated porcine rotavirus vaccines regain virulence and cause clinical transmission. Overall, live attenuated vaccines offer a viable option for the immunoprophylaxis of Group A porcine rotavirus, capable of inducing both mucosal and systemic immunity and facilitating large-scale vaccination. They are particularly suitable for immunization programs aiming to enhance the transfer of maternal antibodies by mimicking natural routes of infection.

### 5.2 Inactivated vaccines

Inactivated vaccines against porcine Group A rotavirus are produced by using chemical or physical methods to render intact viral particles non-infectious and incapable of replication. Common inactivating agents include formaldehyde and *β*-propiolactone, which eliminate viral replicative activity by cross-linking viral proteins and nucleic acids (72–75). Based on manufacturing processes and the antigenic composition of the final product, inactivated vaccines can be further classified into whole-virus inactivated vaccines and lysate-based inactivated vaccines; the latter are currently primarily in the experimental research stage (76, 77).

Whole-virus inactivated vaccines retain a relatively intact viral capsid structure. When administered to sows, they induce the production of highly effective serum neutralizing antibodies, which are efficiently transferred to newborn piglets via colostrum and milk, thereby providing passive immune protection (72, 78). However, because whole-virus inactivated vaccines contain a significant amount of viral lipids and nucleic acid components, transient local reactions at the injection site or transient fever were observed in some regions during early application, primarily due to the pyrogenicity of viral envelope lipids and certain byproducts (79, 80). To reduce adverse reactions, researchers have attempted to moderately separate viral capsid proteins from lipid components through methods such as treatment with detergents, preparing a lysate vaccine similar to influenza lysate vaccines, which can improve injection tolerance to some extent (79, 81, 82).

Although inactivated porcine Group A rotavirus cannot replicate, its fully or partially assembled capsid proteins can still be recognized by the immune system, stimulating the production of protective antibodies (3, 83, 84). Currently widely used commercial inactivated porcine rotavirus vaccines are mostly trivalent inactivated vaccines containing porcine transmissible gastroenteritis virus and porcine epidemic diarrhea virus, with the rotavirus component generally selected from the major locally circulating G/P genotype strains. Such inactivated vaccines can effectively boost the immune response when administered to sows that have previously been exposed to the corresponding rotavirus serotypes; however, for piglets born to special non-pathogen-specific sows that have never been exposed to rotavirus, maternal antibodies provided solely by the inactivated vaccine are sometimes insufficient to completely prevent infection and viral shedding. This is primarily because the inactivation process may result in the loss of certain viral components (such as double-stranded RNA) (48, 68, 85). Viral-derived molecules such as double-stranded RNA act as pathogen-associated molecular patterns that can enhance adaptive immune responses by activating innate immune pathways, such as Toll-like receptors; their reduction in inactivated vaccines may result in relatively insufficient immunogenicity, which is why inactivated vaccines are often used in combination with adjuvants to compensate for this deficiency (84, 86–88). To enhance protection for sows and piglets, some manufacturers have introduced inactivated vaccines with increased antigen content or formulations containing innovative adjuvants, which have demonstrated significant advantages in elevating serum and colostrum neutralizing antibody titers (44, 56, 72).

### 5.3 Recombinant subunit vaccines

Recombinant subunit vaccines produce virus-specific protein antigens using recombinant protein expression systems, eliminating the need for large-scale cultivation of live viruses. Recombinant subunit vaccines for porcine Group A rotavirus primarily use genetically engineered capsid proteins VP4, VP6, VP7, or the truncated VP8* protein as antigens. These proteins play a key role in inducing neutralizing antibodies and cross-protective T-cell responses, while avoiding the introduction of irrelevant viral components and nucleic acids that may be present in whole-virus vaccines (56, 89). Because other viral components are removed, subunit vaccines typically exhibit lower reactogenicity than whole-virus or lysate vaccines; however, they often have weaker immunogenicity and frequently require co-administration with potent adjuvants to enhance the immune response (90–92).

Recombinant VP4, VP6, or VP7 protein antigens produced using a baculovirus-insect cell expression system represent a typical development pathway for porcine rotavirus subunit vaccines. Existing studies have shown that although the recombinant VP6 protein cannot induce neutralizing antibodies, it can mediate a certain degree of cross-protection between different G/P genotypes by activating CD4^+^ T-cell responses (93, 94). Multivalent subunit vaccine candidates containing recombinant VP8* proteins from multiple genotypes can induce high-titer neutralizing antibodies against the corresponding genotypes in sows, which are then transferred to piglets via colostrum (48, 89, 95). Another advantage of recombinant subunit vaccines lies in their production process, which completely bypasses the viral amplification steps typically involving chicken embryos or cell culture. This eliminates the risk of residual ovalbumin and other exogenous biological factors while facilitating rapid adjustment of antigen combinations to address the emergence of new prevalent genotypes (87, 96). Since 2020, several recombinant protein vaccines targeting porcine Group A rotavirus have successively entered clinical trials and field efficacy evaluation phases. Some candidate vaccines have already shown a trend of being comparable to or superior to commercial inactivated vaccines in reducing the incidence of diarrhea and the intensity of viral shedding in piglets (15, 56).

### 5.4 Multivalent swine Group A rotavirus vaccines

Due to the extremely complex serotypic and genotypic diversity of swine Group A rotavirus, and the limited cross-protection between different G and P types, vaccine formulations must contain antigens from multiple dominant circulating strains to achieve broad protection. Consequently, commercial porcine rotavirus vaccines are often formulated as bivalent, trivalent, or multivalent vaccines, containing antigenic components from multiple G/P genotype combinations. The selection of these strains is typically based on regional molecular epidemiological surveys to cover the genotypes currently prevalent in swine populations (10, 22, 62, 63, 97).

Taking the U.S. market as an example, approved inactivated porcine rotavirus vaccines often contain several G-type antigens, such as G4, G5, and G9; in China, the rotavirus components in commercial trivalent or bivalent live and inactivated vaccines are primarily designed based on dominant local strains (e.g., G5, G9). As new genotypes gradually increase in pig populations, vaccine formulations may be adjusted accordingly (98). For example, in recent years, the detection rates of relatively rare genotypes such as G26 and G11 have increased in pig populations in certain regions, and relevant strains have begun to be included in the screening of candidate vaccine strains (7, 99–101). At the same time, dynamic monitoring of the prevalence of specific genotypes in pig populations has also led to a trend toward more streamlined and precise compositions of multivalent vaccines. Long-term monitoring data indicate that if a particular genotype remains undetected in major endemic regions over an extended period, its component may be considered for removal from multivalent vaccines in the future to streamline production processes and reduce vaccine costs (3, 62, 65, 102). However, unlike influenza vaccines, which update their strain formulations annually, the frequency of strain adjustments for porcine rotavirus multivalent vaccines is relatively low; decisions to reformulate typically require epidemiological data and efficacy evaluation results spanning several years.

Overall, the aforementioned types of porcine Group A rotavirus vaccines have demonstrated varying degrees of efficacy in reducing clinical diarrhea in piglets and lowering viral shedding. Inactivated whole-virus vaccines are the most widely used in commercial applications due to their balance of immunogenicity and safety; live-attenuated vaccines, with their advantages of inducing intestinal mucosal immunity and facilitating oral administration, are suitable for specific immunization protocols; recombinant subunit vaccines, on the other hand, eliminate the need for handling live viruses and inactivation steps, demonstrating faster production adaptability and higher safety. Key factors determining the field protective efficacy of vaccines include the degree of genotypic match between the vaccine strain and circulating strains, the immune status of sows, the level of maternal antibodies in piglets, and adjuvant strategies. Building on this, a multivalent vaccine strategy which effectively broadens the spectrum of immune protection by rationally combining antigens from dominant circulating strains is currently the primary approach to addressing the high genetic diversity of porcine Group A rotavirus (10, 22, 63).

## 6 Efficacy of approved vaccines

The field protective efficacy of swine Group A rotavirus vaccines is typically evaluated through observational cohort studies or challenge trials. Key evaluation indicators include the incidence of diarrhea in newborn piglets, the severity of diarrhea, viral shedding levels, and mortality rates, as well as the correlation between specific neutralizing antibody titers in colostrum and milk from immunized sows and the rate of passive protection in piglets (15, 56, 103, 104). Similar to influenza vaccines, the efficacy of porcine rotavirus vaccines also exhibits significant variability depending on the farm, the genotype of the circulating virus, the immune status of sows, and the level of maternal antibodies in piglets (105). The key factor determining protective efficacy is the degree of G/P genotype match between the vaccine strain and the field-circulating strain; when the genotypes are identical or highly similar, the vaccine typically reduces the incidence of diarrhea in piglets by 60–80% or more, whereas cross-protection is often limited on farms with poor genotype matching (10, 63).

Even if the vaccine strain and the circulating strain belong to the same G or P genotype, the gradual accumulation of point mutations within key neutralizing epitope regions on the VP7 and VP4 proteins (i.e., antigenic drift) can still lead to a significant decline in neutralizing antibody titers (6, 64, 106). For example, within the G9 genotype, amino acid substitutions in the VP7 protein among different sublineages can significantly alter its antigenicity, resulting in reduced neutralizing activity of inactivated vaccines prepared from early G9 strains against recent G9 variants (107–109). Furthermore, the segmented genome of porcine Group A rotavirus leads to frequent reassortment; when only one of the G or P types of a field strain matches the vaccine strain, the cross-protective effect is typically weaker than when both G and P types match. This situation bears similarities to the reduced efficacy of the H3N2 component in human influenza vaccines due to antigenic drift and adaptation to chicken embryos; however, the primary driver of reduced efficacy in porcine rotavirus vaccines stems from the genetic diversity of the field strains themselves, rather than adaptive mutations during the production process (10, 70, 110, 111). In recent years, as the prevalence of previously rare genotypes such as G26 and G11 has increased in some regions, multivalent vaccines formulated against traditional circulating strains such as G4, G5, and G9 have demonstrated very limited cross-protection against these emerging genotypes. Some pig farms have continued to experience high rates of piglet diarrhea and mortality even after detecting novel reassortant strains (4, 12, 99).

Vaccine efficacy also varies significantly depending on the target population and immunization protocol. In the classic “sow vaccination colostrum transfer passive protection of piglets” protocol, the level of protection conferred to piglets is highly dependent on the titers of rotavirus-specific IgA and neutralizing antibodies in colostrum, as well as the timing and volume of colostrum ingested by the piglets (112). For sows with a clear immunization history who have completed the recommended prenatal booster schedule, the piglets they produce typically receive robust protection against matched strains during the peak period of maternal antibody transfer; however, in piglets from sows with inadequate primary immunization or those with insufficient colostrum intake, the protective effect is significantly reduced (112). In contrast, although oral administration of live attenuated vaccines to piglets can induce an active mucosal immune response, the immunogenicity of these vaccines is susceptible to interference from maternal antibodies; when high levels of passively acquired antibodies are present in the piglet, the intestinal colonization and immunogenicity of the live vaccine may be suppressed (113, 114).

To enhance vaccine efficacy, inactivated vaccines with increased antigen content and vaccine formulations containing novel adjuvants have been developed for sows with weak immune responses or for seasonal outbreaks with poor antigenic match (72). The addition of oil-based or polymer adjuvants to inactivated vaccines can significantly increase the levels of rotavirus-specific IgA and neutralizing antibody titers in sows’ colostrum, thereby providing effective passive immune protection to piglets. Research by Tang et al. demonstrated that colostrum neutralizing antibody titers were significantly elevated in immunized sows; following challenge, only a few piglets exhibited mild diarrhea, whereas all piglets in the unvaccinated challenge group became ill. Additionally, histopathological lesions in the intestinal tissues of piglets in the immunized group were significantly reduced, and viral shedding was markedly decreased (56). Systematic monitoring data show that vaccinating sows with a multivalent inactivated vaccine containing antigens from currently dominant circulating strains can induce high levels of serum neutralizing antibodies and efficiently transfer maternal antibodies to piglets, significantly reducing the severity of diarrhea in piglets following challenge and substantially lowering viral shedding levels. A meta-analysis further indicates that when the G/P genotypes of field-circulating strains and vaccine strains show a high degree of match, the vaccine provides significant protection; however, when genotype matching is poor, the protective effect decreases markedly. Therefore, in years or regions where the major circulating strains show high genotypic match with the vaccine strain, vaccination of sows with a multivalent inactivated vaccine can significantly reduce the population incidence of rotavirus diarrhea in newborn piglets; however, on farms where new dominant reassortant strains emerge, vaccine efficacy is greatly diminished due to reduced genotypic match (65, 105). Current research evidence indicates that when the G/P genotypes of field-circulating strains remain matched with the vaccine strain, inactivated vaccines can maintain relatively stable protective efficacy over multiple reproductive cycles (10). However, because the segmented genome of rotavirus allows for reassortment of the VP4 and VP7 genes between different strains, vaccine efficacy may be significantly weakened once a new dominant circulating strain emerges (56). When no significant antigenic variation occurs in the field, the incidence of diarrhea in piglets can be maintained at a low level; however, when new dominant strains (especially reassortant strains) are introduced, the vaccine’s protective efficacy may decline rapidly.

The impact of booster vaccination strategies on vaccine efficacy has also drawn attention in the prevention and control of porcine Group A rotavirus. Unlike human influenza vaccines, which require annual updates to their strain composition based on forecasts, the strain composition of porcine rotavirus vaccines is adjusted less frequently. It is currently standard practice to administer an inactivated vaccine booster to sows prior to each farrowing. Most studies indicate that regular booster immunizations can maintain high titers of neutralizing antibodies in colostrum, and no significant immune interference effects resulting from repeated vaccinations have been observed (3, 90, 115, 116). However, limited data suggest that when the same vaccine formulation containing the same adjuvant is used frequently for boosters within a short period, the increase in antibody titers may gradually plateau, indicating room for optimization of the immunization schedule (90, 117). The general consensus is that, under current conditions, a strategy of prepartum vaccination for sows with each litter can effectively reduce the prevalence and severity of diarrhea in piglets in the farrowing house; even if the vaccine does not provide complete protection against infection, it can still generate significant economic benefits by reducing viral shedding and shortening the duration of illness. Future research priorities include: designing universal vaccines based on conserved antigens (such as the VP6 protein or VP8* fusion protein) in combination with novel adjuvants capable of inducing cross-neutralizing antibodies and mucosal immunity, to achieve broader and more durable cross-protection against multiple G/P genotypes.

### 6.1 Correlates of protective immunity against porcine RVA

Understanding immune correlates of protection is critical for rational vaccine and adjuvant design. For porcine RVA, three key immune effectors are linked to protective efficacy, with varying roles in passive and active immunization scenarios:

#### 6.1.1 Serum and colostral neutralizing antibodies

Neutralizing antibodies targeting VP7 and VP4 are the most well-established correlate of protection against homologous strains. In the classic sow immunization–passive protection model, the titer of rotavirus-specific neutralizing antibodies in colostrum is directly correlated with the degree of protection in suckling piglets; higher titers typically correspond to reduced diarrhea incidence, shorter disease duration, and lower viral shedding (112). This is the primary mechanism by which inactivated vaccines confer protection, as they efficiently boost serum IgG that transfers to colostrum. However, neutralizing antibodies are highly genotype-specific, and their protective efficacy drops sharply against heterologous G/P strains.

#### 6.1.2 Intestinal secretory IgA (sIgA)

Intestinal mucosal sIgA acts as the first-line immune barrier against enteric RVA infection by blocking viral attachment to epithelial cells and mediating intracellular neutralization (53). Live attenuated vaccines administered via the oral route induce robust intestinal sIgA responses by mimicking natural infection, which correlates with reduced viral colonization and shedding (10, 69). For heterologous strains, non-neutralizing sIgA targeting conserved viral proteins (e.g., VP6) can also mediate cross-protective effects, though the magnitude of this protection is generally weaker than that of homotypic neutralizing antibodies. Adjuvants that enhance mucosal homing and sIgA class switching are therefore particularly valuable for improving both homologous and heterologous protection.

#### 6.1.3 Virus-specific T-cell responses

CD4^+^ helper T cells support B-cell antibody production and affinity maturation, while CD8^+^ cytotoxic T cells clear virus-infected intestinal epithelial cells. T-cell responses, particularly those targeting conserved internal proteins such as VP6, are considered the primary mediators of heterologous cross-protection, as they are less affected by G/P genotype variation (118). Studies in pig models have shown that strong T-cell responses correlate with reduced intestinal pathology and faster viral clearance even when neutralizing antibody titers are low (55, 60). However, T-cell immunity typically does not prevent initial infection, and there are currently no standardized quantitative correlates of T-cell-mediated protection for field application.

Collectively, optimal vaccine performance requires a balanced induction of neutralizing antibodies, mucosal sIgA, and cellular T-cell responses. Different vaccine platforms and adjuvant strategies differ substantially in their ability to induce these three arms of immunity, which explains their varying protective profiles against homologous and heterologous strains.

## 7 Production time

The production cycle for swine Group A rotavirus vaccines varies significantly depending on the technological platform used. For inactivated cell-culture vaccines the most widely used type the process typically takes 4–6 months, encompassing vaccine strain selection, adaptation to MA-104 or Vero cells, large-scale cultivation, inactivation treatment, multivalent formulation, and final product testing (119–121). This extended timeline is primarily constrained by the adaptive replication of the virus in cells, validation of the inactivation process, and stability assessments following the formulation of the multivalent vaccine (121, 122). The production of live attenuated vaccines also relies on cell culture amplification and requires additional attenuation passaging or construction of reassortant strains; the overall production cycle is similar to or even longer than that of inactivated vaccines (121, 123, 124). Compared to the traditional methods described above, subunit vaccines based on recombinant protein expression can significantly shorten production time. By directly producing protective antigens such as VP7, VP4, or VP8* through baculovirus-insect cell or mammalian cell expression systems, there is no need for large-scale live virus culture and inactivation. The upstream protein expression stage can be completed within a few weeks, making updates to vaccine antigen components more flexible and enabling a faster response to changes in the genotypes of circulating strains (89, 96, 125, 126). This accelerated production model offers distinct advantages for addressing important emerging reassortant strains in pig populations; however, the administrative approval process for veterinary vaccines and large-scale production capacity remain key factors limiting their widespread application.

## 8 Limitations of swine Group A rotavirus vaccines

While existing swine Group A rotavirus vaccines have played a significant role in reducing diarrhea-related morbidity and mortality in piglets, several key limitations remain that undermine their sustained and stable protective efficacy. First, vaccine design relies heavily on molecular epidemiological surveillance and prediction of dominant G/P genotypes in endemic regions. However, porcine Group A rotavirus continuously generates new antigenic variants through genetic reassortment and the accumulation of point mutations, often leading to a decline in antigenic match between vaccine strains and field-circulating strains (1, 10). Second, most currently available commercial mainly primarily target the viral capsid proteins VP7 and VP4, which are the major neutralizing antigens for the G and P types, respectively; however, the genes encoding these proteins are highly variable, and the resulting immune response exhibits significant genotype specificity. This narrow immunological targeting results in insufficient cross-protection against heterologous strains or newly emerging reassortant strains; even when sows receive timely prenatal booster vaccinations, it may still be difficult to completely prevent outbreaks of diarrhea in suckling piglets caused by mismatched strains (12, 63). A meta-analysis of multicenter observational studies indicates that the protective efficacy of human rotavirus vaccines exhibits a marked gradient depending on the degree of match between circulating and vaccine strains. Under conditions of high match, the vaccine’s efficacy against homologous strains remains high (e.g., in high-income countries, the combined efficacy against homologous strains can reach 94%); however, when antigenic differences arise, efficacy against partially or fully heterologous strains drops significantly (e.g., efficacy against some heterologous strains may fall to 71%). In the field of porcine rotavirus vaccines, the cross-protection of currently available commercial G5 vaccines against other circulating genotypes is similarly limited. The field efficacy of vaccines varies significantly across different pig farms and years, with the core limiting factor being the degree of G/P genotype match between the vaccine strain and the field-circulating strain (12, 105). Furthermore, the characteristics of the immune response in specific pig populations also limit the full realization of vaccine efficacy: the immune systems of newborn piglets are immature, and their protection relies entirely on maternal antibodies passively acquired through colostrum and milk. However, maternal antibody levels are influenced by various factors, including the sow’s vaccination regimen, body condition, and the piglet’s colostrum intake, leading to inconsistent protective outcomes; simultaneously, high levels of maternal antibodies may interfere with the intestinal colonization of orally administered live attenuated vaccines and the establishment of an active immune response in piglets (113, 114, 127).

This maternal antibody interference represents a well-documented and pervasive practical challenge in rotavirus vaccine management across both human and veterinary medicine. As comprehensively summarized by Otero et al. (114), maternally derived antibodies are transmitted to offspring through two distinct pathways: transplacental transfer of IgG into the systemic circulation, and breast milk delivery of secretory IgA to the gastrointestinal tract. Multiple mechanisms underlie this interference effect: maternal antibodies can directly neutralize live attenuated vaccine virus and suppress its replication in enterocytes; they can mask key antigenic epitopes and prevent recognition by neonatal B cells; and they can cross-link B cell receptors with inhibitory Fcγ receptors to downregulate B cell activation and differentiation. In commercial swine production systems, this issue is especially pronounced for orally administered live attenuated vaccines: high titers of rotavirus-specific IgA in colostrum and milk persist in the intestinal lumen of suckling piglets, restricting vaccine virus replication and blunting the induction of both mucosal and systemic active immunity. This maternal antibody effect is a major contributor to the well-documented gap between vaccine efficacy measured in controlled antibody-free pig models and actual protective performance in conventional commercial herds.

Beyond immunological barriers, multiple practical implementation challenges further shape the real-world performance of porcine RVA vaccines in commercial pig production. First, regional differences in circulating RVA genotypes directly affect the antigenic match and protective efficacy of available vaccines. For instance, G5 and G9 genotypes currently dominate in Chinese swine populations, whereas G4 and G5 are more prevalent in North American herds, and rare genotypes such as G11 and G26 have been increasingly detected in multiple regions worldwide in recent years. This geographic heterogeneity means that a vaccine formulation demonstrating strong efficacy in one region may exhibit substantially reduced protection in another, necessitating dynamic adjustment of vaccine strain composition based on continuous local molecular epidemiological surveillance.

Second, field efficacy is consistently lower than efficacy measured in controlled experimental challenge trials. Experimental studies are typically conducted under highly standardized conditions with defined challenge doses, uniform piglet health status, and controlled environmental parameters. In contrast, commercial farms face complex real-world confounding factors including co-infections with other enteric pathogens such as porcine epidemic diarrhea virus (PEDV), transmissible gastroenteritis virus (TGEV), and pathogenic *Escherichia coli*, variable parity and pre-existing immune status of sows, and inconsistent farm management and biosecurity levels — all of which can attenuate the measurable protective effect of vaccination.

Third, the operational feasibility of different immunization protocols constrains practical deployment. While intranasal or oral mucosal vaccination offers clear immunological advantages for an enteric pathogen, these routes require more precise operational execution and are more susceptible to environmental interference, making large-scale implementation in intensive pig production systems more challenging than conventional intramuscular injection. Additionally, the strict cold-chain requirements of live attenuated vaccines increase logistical difficulty and deployment costs, particularly in remote or resource-limited farming regions.

In terms of production processes, the continuous adaptation and amplification of the virus on passage cells such as MA-104 during the preparation of inactivated vaccines may lead to changes or loss of certain epitopes on the viral capsid proteins, thereby affecting the antigenic match between the final vaccine product and field-circulating strains (64, 128, 129). Furthermore, traditional intramuscular administration of inactivated vaccines predominantly induces a serum antibody response, with limited capacity to induce secretory IgA in the intestinal mucosa, making it difficult to establish an effective first-line immune barrier at the viral entry sites (130, 131).

**Figure 1:**
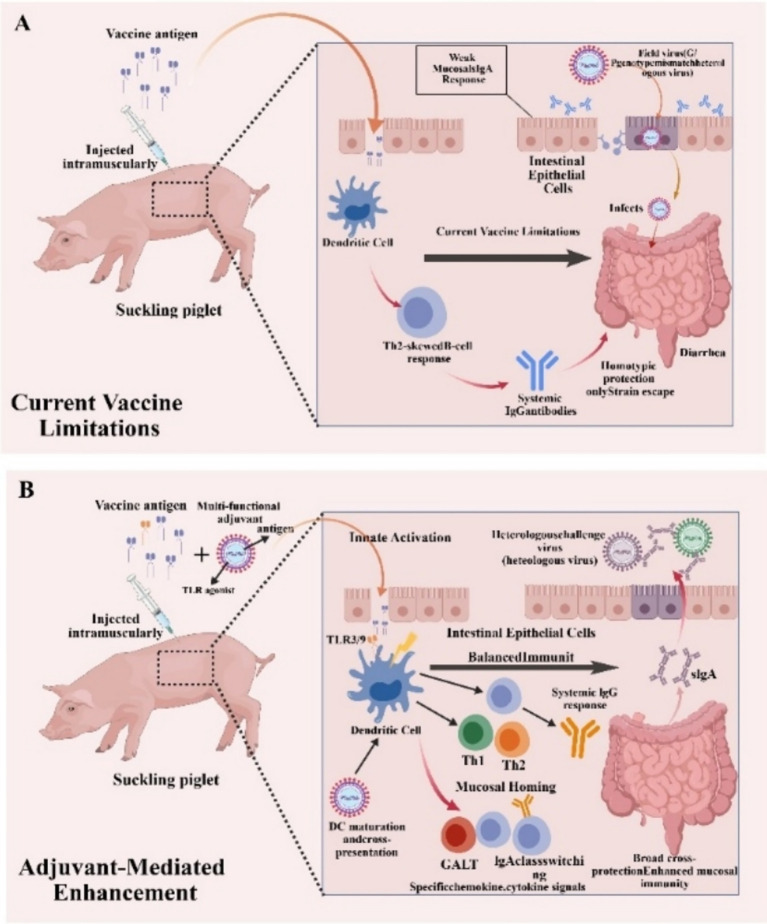
Limitations of conventional swine Group A rotavirus vaccines and the mechanisms by which adjuvants enhance protective immunity. **(A)** Conventional vaccines induce mainly systemic IgG but poor intestinal sIgA, failing to block heterologous rotavirus infection. **(B)** Adjuvants (e.g., nanoparticles + TLR agonists) activate innate immunity, promote balanced Th1/Th2 responses, and generate gut-homing sIgA, enabling broad cross-protection against multiple G/P genotypes.

Taken together, these limitations highlight a fundamental mismatch between the evolutionary characteristics of porcine RVA high genetic diversity, rapid antigenic variation, and mucosal tropism and the immune response profile induced by conventional vaccines, which is dominated by genotype-specific systemic IgG. This mismatch underscores the urgent need to develop next-generation swine Group A rotavirus vaccines capable of providing broad cross-protection, long-lasting immunity, and effective stimulation of intestinal mucosal immunity, as visually summarized in Figure 1.

As illustrated in Figure 1, conventional intramuscular vaccines (Panel A) rely almost entirely on systemic IgG production and fail to induce robust intestinal mucosal sIgA, leaving the primary site of viral invasion unprotected. At the same time, the narrow genotype specificity of these antibody responses means they cannot effectively neutralize heterologous strains with mismatched G/P genotypes, allowing viral escape and clinical disease. In contrast, adjuvant-enhanced vaccine formulations (Panel B) address both gaps simultaneously: by activating innate immune signaling pathways such as Toll-like receptors, they promote a balanced Th1/Th2 immune response and enhance cellular immune function, compensating for the limited protection of humoral immunity alone against variant strains; by inducing gut-homing of immune cells and sIgA class switching in mucosal-associated lymphoid tissues, they establish a local immune barrier at the intestinal epithelium to block viral entry at its source. This mechanistic complementarity explains why adjuvant optimization has emerged as the most feasible and cost-effective strategy to improve the performance of existing vaccines without modifying their antigen composition.

To systematically integrate the performance characteristics of existing vaccine platforms and provide a translational reference for both swine clinicians and vaccine researchers, the key dimensions of four major vaccine categories including immunogenicity, duration of protection, breadth of heterologous cross-protection, safety profile, production barriers, and commercial applicability are summarized and compared in Table 2. This comparative analysis reveals a clear core trade-off: established commercial vaccine platforms benefit from mature production processes, well-characterized safety profiles, and widespread field acceptance, but are inherently constrained by genotype-dependent protective efficacy. In contrast, next-generation universal vaccine strategies hold the promise of broad cross-genotype protection, but face technical, regulatory, and economic barriers to large-scale field deployment. This trade-off further reinforces the central role of adjuvant strategies as a transitional and complementary solution that can enhance the breadth and durability of protection while leveraging existing vaccine production infrastructure.

**Table 2:** Summary of vaccine platforms and adjuvant strategies for porcine Group A rotavirus.

Category	Specific type	Primary mechanism of action	Level of supporting evidence	Key limitations	References
Vaccine platforms	Live attenuated vaccines	Limited intestinal replication induces mucosal sIgA and systemic immunity	Homologous protection well-established in field use; partial cross-protection against closely related strains	Genotype-dependent efficacy; requires cold chain; theoretical reversion risk	(10, 71)
	Inactivated whole-virus vaccines	Induces high-titer serum neutralizing antibodies for maternal transfer	Homologous protection well-documented in commercial settings; weak heterologous efficacy	Poor mucosal sIgA induction; dependent on adjuvants for optimal response	(72, 86)
	Recombinant subunit vaccines	Targeted VP4/VP7/VP6 antigens induce specific antibody and T-cell responses	Homologous immunogenicity confirmed; limited heterologous challenge data	Weak intrinsic immunogenicity; requires potent adjuvant formulations	(56, 89)
	Universal vaccine approaches (VP6, mRNA, multi-epitope, chimeric)	Target conserved viral regions to induce cross-genotype immunity	Proof-of-concept in preclinical pig/small animal models; no commercial field data	Unestablished protective correlates; manufacturing complexity; high cost	(45, 206)
Adjuvant strategies	Aluminum salts	Antigen adsorption depot; NLRP3 inflammasome activation	Confirmed enhanced homologous immunogenicity; minimal heterologous benefit	Th2-skewed response; poor mucosal immunity induction	(143, 149)
	Oil-in-water / water-in-oil emulsions	Sustained antigen release; recruitment of innate immune cells	Improves homologous protection; partial benefit against partially mismatched strains	Local injection site reactions; limited mucosal sIgA enhancement	(72, 135)
	Saponins (Quil A, ISCOMs)	Enhanced antigen cross-presentation; balanced Th1/Th2 response	Improved homologous and partial heterologous protection	Local reactogenicity at high doses; complex manufacturing	(165, 168)
	TLR agonists (poly(I:C), CpG ODN, MPL)	Activate innate pattern recognition receptor pathways	Enhanced cellular and mucosal immunogenicity; preliminary cross-protection signal from other viral models	Limited direct porcine RVA challenge data; variable stability *in vivo*	(61, 137)
	Nanoparticle carriers (chitosan, PLGA)	Mucosal targeting; sustained antigen release; enhanced uptake	Improved mucosal immunogenicity; partial heterologous protection in preclinical models	Scalability challenges; batch-to-batch variability	(57, 186)
	Combination adjuvant systems	Synergistic activation of multiple immune pathways	Strongest preclinical cross-protection signal	Formulation complexity; safety and cost barriers to field deployment	(191, 192)

## 9 Vaccine adjuvants

Building directly on the limitations of conventional vaccines outlined above genotype-restricted protective efficacy, insufficient intestinal mucosal immunity, and susceptibility to maternal antibody interference adjuvant strategies provide a targeted and technically feasible solution that does not require altering the antigen composition of existing vaccine products. An adjuvant is a substance added to a vaccine formulation or administered alongside an antigen to enhance the specific immune response; it is particularly important for inactivated and subunit vaccines. In the context of swine Group A rotavirus vaccines, adjuvants also play a critical role: by enhancing the immune response against both matched strains and antigenic variants, adjuvants help broaden the spectrum of protection and reduce the decline in efficacy caused by antigen mismatch (132–135). Adjuvants can enhance the intensity and duration of the immune response, thereby reducing the required antigen dose while achieving protective immunity (dose-sparing effect) and improving the level of immune protection in populations with weaker immune responses (such as primiparous sows and newborn piglets) (48, 135, 136). These effects are achieved through various mechanisms, including the regulation of innate immune pathways and the formation of antigen reservoirs at the injection site.

When evaluating adjuvant performance in porcine RVA vaccine development, it is critical to distinguish three hierarchical levels of evidence:

1. Enhanced immunogenicity: Elevated antigen-specific antibody titers or cellular immune responses, measured without viral challenge. This is the most commonly reported endpoint in early-stage adjuvant studies but does not guarantee improved protective efficacy.
2. Improved homologous protection: Reduced diarrhea severity and viral shedding following challenge with a strain genetically matched to the vaccine antigen.
3. Confirmed heterologous cross-protection: Significant disease reduction following challenge with strains bearing mismatched G/P genotypes.

While most adjuvant strategies have demonstrated enhanced immunogenicity, the extent to which they can overcome genotype mismatch and confer broad heterologous protection remains incompletely established. The following sections contextualize each adjuvant class against this evidence framework.

### 9.1 Mechanism of action of adjuvants

Adjuvants initiate and enhance the immune response to vaccines through various complementary mechanisms, including activation of the innate immune system, enhancement of antigen presentation, and modulation of the adaptive immune response. Through these mechanisms, adjuvants result in higher antibody titers, a broader immune profile, and longer-lasting protection (135).

Activation of innate immune receptors: Various adjuvants can activate pattern recognition receptors such as Toll-like receptors (TLRs) and inflammasomes, thereby activating intracellular signaling pathways such as NF-κB and MAPK, and promoting the production of chemokines and pro-inflammatory cytokines. These molecules create a local inflammatory microenvironment at the injection site, recruiting immune cells and directing them to regional lymph nodes (137, 138).

Regulation of Antigen Presentation and Uptake: Adjuvants enhance the uptake of co-administered antigens by antigen-presenting cells (APCs), such as dendritic cells and macrophages. They promote the maturation of APCs and upregulate the expression of major histocompatibility complex (MHC) molecules, thereby improving antigen processing and T-cell activation (139–142).

Reservoir effect and antigen retention: Some adjuvants (particularly aluminum salts and oil-in-water emulsions)form local reservoirs after injection, delaying antigen release and maintaining prolonged antigen exposure in the presence of immune stimulatory signals, thereby extending the duration of the immune response (143–145).

Cytokine Induction: Adjuvants stimulate innate immune pathways to induce the secretion of cytokines such as IL-6, IFN-*γ*, and CCL2. These cytokines support effective B-cell and T-cell responses while promoting both humoral and cellular immunity (146).

Regulation of T Helper Cell Responses: Different adjuvants can steer the immune response toward a Th1 or Th2 phenotype. Th1-type adjuvants enhance cytotoxic T-lymphocyte activity and promote the production of IgG2a/IgG3 antibodies, whereas Th2-type adjuvants tend to induce IgG1/IgE antibodies and assist in T-cell differentiation. Some novel adjuvants are designed to promote a balanced Th1/Th2 response, which is particularly advantageous for antiviral immunity (90, 147–150).

Inflammasome activation: Particulate adjuvants such as aluminum salts can activate the NLRP3 inflammasome in antigen-presenting cells, triggering the production of potent pro-inflammatory cytokines such as IL-1β and IL-18, which further enhance the adaptive immune response (149–154).

In 1926, aluminum salts (particularly potassium aluminum sulfate, i.e., aluminum adjuvant) were introduced as vaccine adjuvants to enhance the immunogenicity of diphtheria toxoid (155). Although the success of aluminum adjuvants spurred subsequent exploration of immunostimulants, aluminum adjuvants remained the only adjuvant approved for human use for most of the 20th century. It was not until the late 1990s and early 2000s that new adjuvants such as MF59, AS01, AS03, AS04, AF03, and viromides were successively approved, some of which have been incorporated into influenza vaccines (145, 155–158). These modern adjuvants offer new mechanisms of action and greater immunological precision, marking a shift in adjuvant R&D from empirical discovery (aluminum adjuvants) to rational design. In the field of veterinary vaccines, the evolution of adjuvants for swine vaccines has followed a similar trajectory: traditionally dominated by aluminum salts and mineral oil emulsions, recent years have seen increasing research on novel adjuvants such as TLR agonists, nanoparticles, and saponins in porcine rotavirus vaccines, driven by a deeper understanding of the need for intestinal mucosal immunity and cross-protection in swine (135, 159).

### 9.2 Aluminum salt adjuvants

Aluminum salts such as aluminum hydroxide and aluminum phosphate (collectively referred to as aluminum adjuvants)have been used as vaccine adjuvants for over 70 years (160). Aluminum adjuvants enhance the immune response through the following mechanisms: (i) by adsorbing vaccine antigens through the formation of insoluble aggregates, thereby presenting the antigens in a multivalent form that facilitates binding and uptake by antigen-presenting cells (161); (ii) by activating the NOD-like receptor protein 3 (NLRP3) inflammasome, creating an inflammatory environment at the injection site, promoting dendritic cell maturation and T helper cell activation, and ultimately enhancing the adaptive immune response against the co-injected antigen (151, 162). In swine Group A rotavirus vaccines, aluminum adjuvants are primarily used in inactivated vaccines and can significantly increase the titers of rotavirus-specific IgG and neutralizing antibodies in the serum of immunized sows. However, aluminum adjuvants tend to induce a Th2-type immune response and have limited ability to induce secretory IgA (sIgA) in the intestinal mucosa; therefore, the amount of protective antibodies transferred to piglets via colostrum is relatively insufficient (76, 143). Studies have shown that when sows are immunized with an aluminum-adjuvanted inactivated porcine rotavirus vaccine, the reduction in diarrhea incidence and viral shedding intensity in their piglets following challenge with a heterologous strain is generally less than that achieved with oil-emulsion-adjuvanted vaccines (72, 76, 149). Furthermore, when aluminum-adjuvanted vaccines are administered orally to newborn piglets, their ability to induce active immunity in the intestinal mucosa is weak. Therefore, although aluminum adjuvants offer the advantages of good safety and low cost, in porcine rotavirus vaccines, they are typically used in combination with oil emulsions or other immunostimulants to achieve more comprehensive intestinal protection and cross-protection (72, 149).

In terms of evidence hierarchy, aluminum adjuvants are well-documented to enhance homologous immunogenicity but provide minimal benefit against heterologous challenge, consistent with their Th2-skewed, antibody-focused mechanism of action (76, 149).

### 9.3 Oil-in-water and water-in-oil adjuvants

Oil-in-water and water-in-oil adjuvants are the most widely used types of adjuvants in swine vaccines, including oil-in-water emulsions and certain water-in-oil emulsions. In inactivated swine Group A rotavirus vaccines, mineral oil adjuvants (such as the Montanide ISA series) can form an antigen reservoir at the injection site, delay antigen release, and recruit macrophages and dendritic cells, thereby enhancing local and systemic immune responses (72, 135). Compared with aluminum adjuvants, oil-in-water emulsions induce a stronger and more persistent antibody response and can induce a more balanced Th1/Th2 immune response, which is conducive to the production of higher titers of neutralizing antibodies and a certain level of intestinal sIgA precursor cells (72, 148). Following prepartum vaccination of sows with an oil-in-water emulsion-based inactivated porcine rotavirus vaccine, the titers of rotavirus-specific IgA and neutralizing antibodies in colostrum and mature milk significantly increased. Newborn piglets acquired effective passive protection against the same viral strain through ingestion of colostrum, and both viral shedding intensity and the duration of diarrhea were significantly reduced following challenge (48, 65). In the design of multivalent vaccines for swine, immunological interference among antigenic components is a common challenge. Oil-in-water or water-in-oil-based emulsified adjuvants, due to their unique sustained-release and immunomodulatory properties, have been shown to support balanced and highly effective immune responses induced by multiple antigenic components in vaccines. In field trials of a trivalent oil-in-water emulsion vaccine targeting three serotypes of *Actinobacillus pleuropneumoniae*, post-vaccination complement-fixing antibody titers showed a significant increase in responses against all three serotypes, peaking 30 days after the second vaccination, demonstrating that this formulation effectively avoids antigen competition and immunological interference commonly encountered in the development of multivalent vaccines, thereby providing effective and balanced immune protection against all components (163). Furthermore, preliminary evaluations of water-in-oil emulsions (such as MF59 analogs) in subunit vaccines against porcine rotavirus have shown that while they can induce high levels of serum IgG, their ability to enhance intestinal mucosal sIgA remains relatively limited (15, 135, 164). Overall, oil-in-water emulsions are the standard adjuvant of choice for current inactivated swine Group A rotavirus vaccines, and their safety and efficacy have been extensively validated in commercial vaccines.

Oil emulsion adjuvants have strong evidence for improving homologous protective efficacy. They also confer partial cross-protection against strains sharing one genotype (G or P) with the vaccine strain, but cannot fully compensate for complete G/P genotype mismatch (48, 65).

### 9.4 Saponin adjuvants

Saponins are a class of natural glycoside compounds extracted from the bark of the South American soapberry tree (*Quillaja saponaria* Molina) that possess potent immunostimulatory activity. Purified saponin fractions (such as Quil A and QS-21) have been used in various veterinary and human vaccines (165–167). In studies on swine Group A rotavirus vaccines, saponins or saponin-containing immunostimulatory complexes (ISCOMs) significantly enhanced the immunogenicity of inactivated or subunit vaccines. When Quil A was mixed with porcine rotavirus antigens and administered to sows, it induced higher levels of specific IgA and neutralizing antibodies in colostrum compared to aluminum adjuvants and provided better cross-protection in heterologous challenge trials with piglets (168–170). The ISCOM matrix promotes antigen uptake by antigen-presenting cells and cross-presentation via the MHC class I/II pathways by assembling antigens, saponins, cholesterol, and phospholipids into particles of approximately 40 nm, thereby simultaneously activating CD8 + and CD4 + T cell responses (171–173). This offers the potential for cross-protection independent of neutralizing antibodies. Furthermore, saponins (such as Quil A), acting as potent adjuvants, have been shown to significantly enhance the immunogenicity of swine Group A rotavirus vaccines, particularly by boosting the intestinal mucosal immune response (174). Although high doses of saponins may induce local tissue reactions or systemic toxicity in pigs, when administered within the recommended dosage range or optimized through ISCOM formulations, they still demonstrate good safety and immunostimulatory effects.

Saponin-based adjuvants show stronger evidence for heterologous benefit compared to aluminum or basic oil emulsions, likely due to their ability to activate both antibody and cellular immune pathways. However, most cross-protection data come from small-scale experimental trials, and field validation remains limited (168, 170).

### 9.5 Bacterial enterotoxin adjuvants and mucosal adjuvants

Given that porcine Group A rotavirus infects via the fecal-oral route and replicates primarily in the intestinal mucosa, inducing robust intestinal mucosal immunity is one of the core objectives of vaccine design. Bacterial enterotoxins such as cholera toxin (CT) and heat-labile enterotoxin (LT), along with their B subunits (CTB and LTB), were among the first molecules demonstrated to possess potent mucosal adjuvant activity (175, 176). CT and LT promote antigen transport across the epithelial barrier by binding to GM1 ganglioside receptors on the surface of intestinal epithelial cells via their B subunits, while simultaneously activating adenylate cyclase through their A subunits, thereby inducing pro-inflammatory cytokine secretion and dendritic cell activation (177, 178). Intranasal immunization of germ-free pigs with attenuated LT mutants (e.g., LT-R192G) in combination with rotavirus antigens significantly enhanced rotavirus-specific sIgA and systemic antibody responses; however, experimental results showed that piglets immunized intranasally with only 2/6-VLP and mLT (LT-R192G) did not acquire significant protective immunity following challenge (130). This suggests that while bacterial enterotoxin adjuvants can enhance immunogenicity, their use alone does not guarantee protection against challenge; future research may require more optimized antigen delivery or combined immunization strategies. Furthermore, building on Agnello et al.’s confirmation of CTB’s feasibility as a mucosal adjuvant for VLP vaccines, combined with Zhao et al.’s evidence that VLP vaccines can induce passive immunity in colostrum in pig models, it can be inferred that intranasal immunization of sows with CTB-conjugated rotavirus VLP vaccines is expected to simultaneously increase sIgA levels in both colostrum and mature milk, as well as the rate of passive protection in piglets (179, 180). However, the use of enterotoxin adjuvants in swine vaccines remains limited by safety concerns (such as residual toxicity and potential effects on intestinal mucosal structure). Current research efforts are focused on developing completely non-toxic CT/LT mutants or subunit adjuvants to eliminate safety risks while maintaining the activity of mucosal adjuvants.

### 9.6 TLR agonists

Toll-like receptor (TLR) agonists directly activate the innate immune system by mimicking pathogen-associated molecular patterns (PAMPs) from viruses or bacteria, making them a key area of focus in the development of adjuvants for porcine rotavirus vaccines.

TLR3 agonist: The double-stranded RNA (dsRNA) analog poly(I:C) mimics the dsRNA produced during rotavirus replication, activating the TLR3 and MDA-5 signaling pathways to induce the production of type I interferons and pro-inflammatory cytokines (181). In studies of other porcine viral vaccines, the TLR3 agonist poly(I:C) has been shown to significantly enhance cellular and mucosal immune responses. Based on this, it is hypothesized that the combined use of poly(I:C) in Group A porcine rotavirus vaccines may also increase virus-specific antibodies in serum and colostrum and enhance the activity of natural killer cells and cytotoxic T cells (61). Although direct challenge data on the use of poly(I:C) as an adjuvant in porcine rotavirus vaccines are currently lacking, cross-virus studies have shown that poly(I:C) as an adjuvant can significantly reduce the intensity of viral shedding following challenge with heterologous strains in a swine influenza model, suggesting that poly(I:C) also has the potential to enhance cross-protection and reduce viral shedding in porcine rotavirus vaccines (170).

TLR9 agonists: Oligodeoxynucleotides (CpG ODNs) containing unmethylated CpG motifs can activate TLR9 and induce a potent Th1-type immune response. CpG ODNs can activate TLR9 and induce a potent Th1-type immune response. Pasternak et al. combined CpG ODNs as TLR9 agonists with antigens for oral immunization of newborn piglets, successfully inducing significant mucosal and systemic immune responses (181). As a TLR9 agonist, CpG ODN has been demonstrated to be an effective adjuvant for animal vaccines. Studies have shown that TLR9 agonists can effectively promote cytokine responses, such as IFN-*γ*, in pigs (159). Furthermore, the VP6 protein of rotavirus can induce cross-protective immune responses against heterologous strains (182). Therefore, combined immunization with CpG ODNs and inactivated porcine rotavirus antigens or recombinant VP6 protein is expected to provide partial cross-protection against challenge with heterologous strains by promoting IFN-γ production and cytotoxic T-cell responses.

TLR4 agonists: When used in combination with oil-in-water emulsions, TLR4 agonists (such as monophospholipid A, MPL) can further enhance the neutralizing antibody titers and antibody affinity of inactivated vaccines through synergistic delivery mechanisms (183). In inactivated rotavirus vaccines, the combination of MPL with lipid adjuvants has been shown in animal models to significantly enhance immunogenicity and protective efficacy; furthermore, in swine models, MPL adjuvants have also demonstrated clear immunostimulatory effects (53, 184). Although research on the combination of MPL and oil-in-water emulsions in Group A porcine rotavirus vaccines remains limited, the above evidence provides strong support for the potential application of this combination adjuvant strategy in porcine rotavirus vaccines.

TLR agonists have demonstrated enhanced cellular and mucosal immunogenicity across multiple porcine viral vaccine models. Preliminary cross-protection data exist for influenza and other viruses, but direct evidence for heterologous protection against porcine RVA remains largely inferential, with few dedicated challenge studies published to date (61, 170).

### 9.7 Nanoparticle adjuvants

Due to their unique physicochemical properties, polymeric nanoparticle carriers serve as highly efficient vaccine delivery platforms, delivering antigens to the mucosal immune system via oral or intranasal routes (185). Chitosan, a cationic, biocompatible polysaccharide with inherent mucosal adhesion, forms nanoparticles that effectively protect encapsulated antigens from degradation. By targeting mucosal M cells, dendritic cells, and macrophages, these nanoparticles enhance antigen uptake and immunogenicity at mucosal sites (186). In porcine models, chitosan nanoparticles encapsulating inactivated viral antigens, following intranasal immunization, have been shown to significantly enhance mucosal secretory IgA (sIgA) and serum antibody responses, as well as induce cytotoxic T-lymphocyte activity (185). Although current data on direct challenge protection against porcine rotavirus are limited, the aforementioned mechanisms and cross-viral research evidence suggest that chitosan nanoparticles show great promise as a mucosal vaccine delivery system in oral and intranasal porcine rotavirus vaccines. Polylactic-co-glycolic acid (PLGA) nanoparticles can control antigen release and induce sustained antibody and T-cell responses following intramuscular or intranasal immunization, demonstrating partial protection against both homologous and heterologous strains in piglets (57). Research has shown that intranasal immunization of piglets with virus-like particles (such as 2/6-VLPs containing VP6) combined with mucosal adjuvants (such as the mutated heat-labile toxin mLT from *Escherichia coli*) can induce intestinal IgA-secreting cells and a systemic antibody response (187). In addition, cationic polysaccharide nanoparticles such as chitosan have been widely used as mucosal vaccine delivery platforms in oral and intranasal vaccine studies in pigs (186). Therefore, the combined use of liposomes and plant-derived cationic nanoparticles (such as *α*-glucan nanoparticles) with TLR agonists for the delivery of recombinant VP8* subunit antigens of porcine rotavirus is expected to induce stronger cross-reactive mucosal sIgA and systemic antibody responses in piglet models, and this direction warrants further exploration. The advantage of nanoparticle adjuvants lies in their ability to serve simultaneously as carriers for both antigen delivery systems and immunostimulants, enabling the co-delivery of antigens and adjuvants to enhance immunogenicity.

Nanoparticle delivery systems show promising preliminary evidence for enhancing both mucosal immunity and partial heterologous protection in preclinical piglet models. However, data are limited to small experimental studies, and the reproducibility and scalability of these effects for commercial production remain to be confirmed (57, 58).

### 9.8 Cytokine adjuvants

Recombinant cytokines act as adjuvants by directly interacting with immune cells to specifically modulate the immune response. Previous studies have shown that recombinant porcine IL-12 can serve as an adjuvant in porcine DNA vaccines to induce cellular immune responses, while recombinant porcine IL-18, when co-formulated with porcine bacterial vaccines, can accelerate the onset of antibody responses (188, 189). Given that IL-12 and IL-18 have been demonstrated to be key promoters of Th1-type immune responses and can enhance virus-specific cellular immunity in intestinal infection models (190), we hypothesize that co-administering recombinant porcine IL-12 or IL-18 with inactivated or DNA vaccines against porcine rotavirus may enhance virus-specific cytotoxic T-lymphocyte activity in pigs and promote intestinal sIgA secretion. This strategy requires further research for validation. Granulocyte-macrophage colony-stimulating factor (GM-CSF), when used as an adjuvant, can enhance the local recruitment and activation of antigen-presenting cells, thereby increasing antibody response levels. Although cytokine adjuvants have demonstrated proof-of-concept efficacy in porcine rotavirus vaccines, their short half-life, high cost, and potential systemic side effects limit their large-scale field application.

### 9.9 Combination adjuvant strategies

To overcome the limitations of single adjuvants, combination adjuvant strategies are gaining increasing attention. The combination of aluminum adjuvants with TLR agonists (such as CpG ODN)can induce a shift toward a Th1-type response, compensating for the limitation of aluminum adjuvants in inducing only Th2 responses; the combination of oil-in-water emulsions and saponins can simultaneously achieve sustained antigen release and potent immunostimulation, enhancing the breadth and durability of the immune response; co-encapsulation of nanoparticles with poly(I:C) or CpG enables synergistic delivery of antigens and adjuvants, inducing a more coordinated mucosal and systemic immune response (191, 192). In the development of swine Group A rotavirus vaccines, combined adjuvant strategies have preliminarily demonstrated the potential for cross-protection against multiple G/P genotype strains, providing an important technical direction for the development of broadly protective swine rotavirus vaccines.

## 10 Safety considerations of adjuvanted swine Group A rotavirus vaccines

Although adjuvants can significantly enhance the immunogenicity and protective efficacy of swine Group A rotavirus vaccines, their introduction must be carefully evaluated for safety and reactogenicity. Overall, currently licensed inactivated and live attenuated rotavirus vaccines for swine have a good safety record under field conditions. However, the inclusion of adjuvants may be associated with a mild to moderate increase in local and systemic reactions, and occasional immune-mediated adverse events have been reported. Therefore, it is essential to balance immunological benefits with safety risks through continuous pharmacovigilance(Table 2).

Water-in-oil emulsions (e.g., mineral oil adjuvants such as the Montanide ISA series) are the most widely used potent adjuvants for inactivated porcine rotavirus vaccines, capable of inducing long-lasting antibody responses. Nevertheless, their safety has been a concern. The most common adverse reactions are local tissue responses at the injection site, including transient swelling, induration, granuloma formation, and in some cases sterile abscesses (72, 79). These local reactions usually resolve spontaneously or become localized over time, but they may affect carcass quality to some extent. In contrast, oil-in-water emulsions and aluminum salt adjuvants generally cause milder local reactions, typically transient pain or tenderness at the injection site (72, 149, 162).

Regarding systemic reactions, a small proportion of sows may experience transient fever, reduced appetite, or lethargy after vaccination with oil-emulsion-adjuvanted products; these signs are self-limiting and require no specific intervention (72, 79). To date, there is no evidence that the adjuvant components in swine Group A rotavirus vaccines cause autoimmune neurological disorders such as narcolepsy, which was reported in humans with AS03-adjuvanted influenza vaccines. Current understanding suggests that such rare events are more likely related to interactions between specific viral antigens and host genetic backgrounds rather than the adjuvant alone (90, 105). The route of administration also influences the safety profile. Intramuscular injection is the recommended route for adjuvanted inactivated porcine rotavirus vaccines, as subcutaneous or intradermal administration tends to cause more local irritative reactions and skin changes such as pigmentation (72, 135). Conversely, the oral administration of commercial trivalent live vaccines (against rotavirus, TGEV, and PEDV) typically does not contain conventional injectable adjuvants, and occasional transient diarrhea or vomiting is attributed to limited intestinal replication of the live vaccine strains rather than to the adjuvant (10, 68). To ensure continued safety after large-scale field use, a robust veterinary pharmacovigilance system is indispensable. Passive surveillance systems such as the adverse event reporting system of the USDA and the European Union veterinary pharmacovigilance database enable early detection of rare or unexpected adverse reactions, facilitating targeted investigations and maintaining producer confidence in vaccine safety (3, 63, 90).

## 11 Exploring strategies for a universal swine Group A rotavirus vaccine

**Figure 2:**
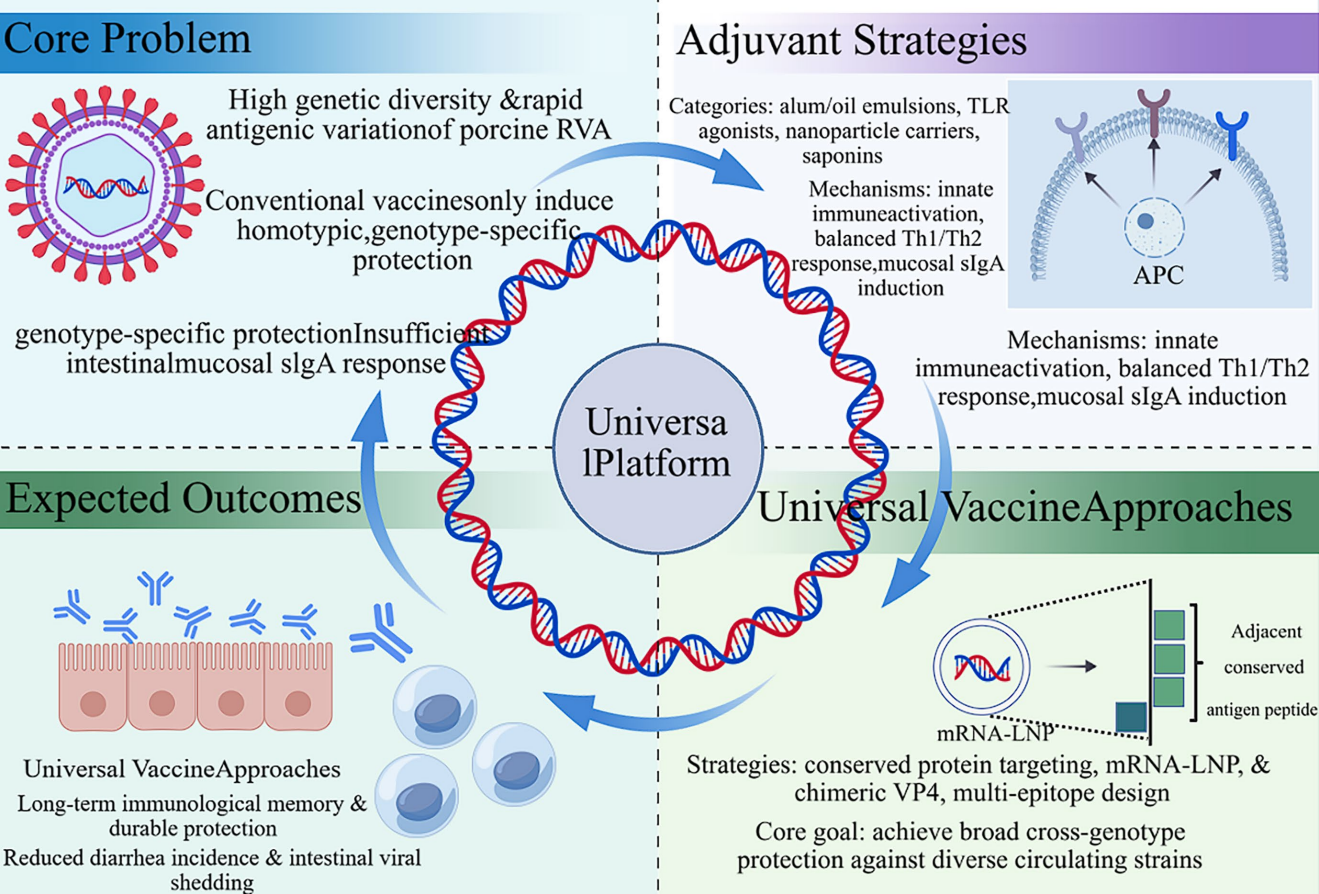
Framework for developing improved swine Group A rotavirus vaccines. This closed-loop diagram illustrates the logical progression of porcine Group A rotavirus (RVA) vaccine optimization. Starting from the core problem of limited homotypic protection caused by high viral genetic diversity, the framework sequentially presents adjuvant strategies as an intermediate solution, universal vaccine approaches as a long-term solution, and the final expected outcomes of enhanced neonatal protection, reduced disease burden and long-term immunological memory.

Currently available commercial swine Group A rotavirus vaccines have played a significant role in reducing the incidence and mortality of diarrhea in piglets. However, because the immune responses they induce are highly G/P genotype-specific, these vaccines struggle to address the constant emergence of new variants driven by antigenic drift and genetic reassortment. To overcome this limitation, the development of a universal swine Group A rotavirus vaccine capable of providing broad and long-lasting cross-protection against multiple G/P genotypes has become a major research focus in this field. Unlike traditional vaccines that primarily target highly variable VP7- and VP4-specific epitopes, universal vaccine strategies aim to direct the immune response toward viral components that are highly conserved across different strains, thereby covering a broader range of circulating variants(Figure 2). The following discusses several strategies that may facilitate the development of a broad-spectrum swine Group A rotavirus vaccine.

### 11.1 Targeting conserved viral proteins

One of the key strategies for overcoming the limitations of existing vaccines is to shift the immunological focus from serotype-specific neutralizing antigens to proteins that are essential for the viral life cycle and are highly conserved in sequence. The VP6 protein is the primary group-specific antigen of Group A rotaviruses, accounting for more than 50% of the total protein content of viral particles, and its amino acid sequence is highly conserved across different G/P genotypes (182). Although anti-VP6 antibodies lack neutralizing activity, intracellular IgA-mediated neutralization has been shown to provide cross-protection across different serotypes. Additionally, VP6 contains multiple conserved epitopes for CD4^+^ and CD8^+^ T cells, which can clear virally infected cells by inducing T-cell responses (118). In suckling rat and newborn piglet models, intranasal or oral administration of a recombinant VP6 protein vaccine significantly reduced viral shedding in the gut and the duration of diarrhea following challenge with heterologous strains; this protective effect primarily relies on T-cell responses and mucosal IgA (193). Furthermore, the VP8* protein, as a trypsin cleavage product of VP4, is responsible for binding to host cell surface receptors (such as HBGAs), and its core domain exhibits a certain degree of conservation across different P-types; neutralizing antibodies targeting this region can exert cross-inhibitory activity against multiple P-type strains (95, 194). The non-structural protein NSP4 possesses enterotoxin activity and contains highly conserved T-cell epitopes; it is also being explored as a candidate antigen for a universal vaccine (195, 196). By co-delivering these conserved antigens such as through the formation of VP6-VP8* fusion proteins or chimeric particles it is hoped that cross-protective antibodies and T-cell responses spanning G/P genotypes can be simultaneously induced. This conserved antigen-based strategy corresponds to the “conserved proteins” branch of the universal vaccine framework shown in Figure 2, representing the most extensively studied and mechanistically validated path toward broad cross-protection.

Despite these promising findings, several critical knowledge gaps remain. First, the quantitative contribution of VP6-specific T cells and intracellular IgA to field-level protection remains poorly defined, and there are no established standardized correlates of protection for these non-neutralizing immune responses. Second, the degree of cross-protection afforded by VP6-based vaccines varies widely across challenge models, and it remains unclear whether this strategy alone can provide sufficient clinical protection against fully heterologous virulent strains under intensive farming conditions. Third, the durability of VP6-induced cellular immunity in growing piglets, and its interaction with maternally derived antibodies ubiquitous in field settings require systematic investigation. Finally, VP6 vaccines typically reduce rather than prevent infection, which may be insufficient for herds pursuing virus elimination.

### 11.2 T-cell vaccine strategies

In swine Group A rotavirus infection, virus-specific T cells particularly memory T cells resident in intestinal tissues play a critical role in clearing the infection and mediating cross-protection. Given that internal proteins such as VP6 contain T-cell epitopes that are highly conserved across the vast majority of swine Group A rotavirus strains, vaccine strategies aimed at inducing potent T-cell responses have a theoretical basis for achieving broad-spectrum protection (197). In mouse and swine models, viral vector vaccines (such as adenovirus or recombinant pseudorabies virus vectors) and DNA vaccines expressing VP6 or the VP6-VP7 fusion protein have been shown to induce significant rotavirus-specific CD4^+^ and CD8^+^ T-cell responses, reducing intestinal viral load and the severity of diarrhea following challenge with heterologous strains (60, 198). However, key challenges facing T-cell vaccines include: the long-term maintenance of memory T cells resident in intestinal tissues depends on limited metabolic reprogramming and external signaling stimulation, the high polymorphism of swine leukocyte antigens (SLA), which significantly affects the efficiency of T-cell epitope presentation and recognition among different individuals; and the fact that maternal antibodies may potentially weaken the T-cell immune response induced by vector vaccines by accelerating the clearance of orally administered live viral vectors (such as adenovirus) (199–201). Current data suggest that, within a T-cell-priming rather than antibody-dependent protective framework, T-cell-targeted vaccines may be particularly valuable for controlling infections caused by new variants; however, further research is needed to optimize immunization regimens and vector design to achieve sustained intestinal mucosal protection.

This T-cell-focused approach represents an important technical direction within the universal vaccine framework outlined in Figure 2, complementing antibody-centered strategies by targeting intracellular stages of viral infection.

Major unresolved challenges limiting the translation of T-cell vaccine strategies include: (1) the limited long-term maintenance of intestinal tissue-resident memory T cells, which may wane rapidly without periodic antigen re-exposure; (2) the high polymorphism of swine leukocyte antigens (SLA), which may lead to highly variable vaccine efficacy across different pig breeds and genetic backgrounds; and (3) potential maternal antibody interference, which may blunt the priming of T-cell responses in neonatal piglets by accelerating vector clearance. Additionally, T-cell-focused vaccines typically reduce disease severity and viral shedding but do not block initial infection, which limits their utility in high-biosecurity breeding systems aiming for complete pathogen exclusion.

### 11.3 mRNA vaccines

Vaccine platforms based on lipid nanoparticle (LNP)-mediated delivery of chemically modified mRNA have demonstrated significant potential in the prevention and control of human infectious diseases, and research into their application in swine rotavirus vaccines is also in its early stages (45). In a trivalent mRNA-LNP vaccine targeting VP8*, a strong VP8*-specific serum IgG antibody response and an IFN-*γ*^+^ T-cell response were simultaneously induced in a germ-free pig model. This significantly reduced the duration and severity of diarrhea following viral challenge and decreased viral shedding, providing new insights for the development of next-generation rotavirus vaccines. Studies have shown that mRNA vaccines encoding conserved rotavirus proteins can induce cross-neutralizing antibodies and T-cell responses against multiple heterologous strains in animal models, and reduce viral shedding levels and the incidence of diarrhea following challenge (45). The mRNA platform possesses intrinsic adjuvant activity, features a short production cycle, and allows for flexible design of antigen sequences, facilitating rapid response to emerging dominant reassortant strains. Furthermore, combination mRNA vaccines capable of simultaneously encoding multiple conserved antigens provide a convenient approach for developing broad-spectrum Group A porcine rotavirus vaccines. Currently, this approach still requires systematic evaluation of immunogenicity and field efficacy in piglets, as well as resolution of practical issues such as production costs and storage stability.

As an emerging platform in the universal vaccine landscape depicted in Figure 2, mRNA vaccines leverage their flexible sequence design to target multiple conserved antigens simultaneously, offering unique advantages for rapid response to viral evolution.

Key practical and scientific barriers remain for clinical translation of porcine RVA mRNA vaccines: (1) production costs at commercial scale are currently substantially higher than traditional inactivated vaccines, limiting economic feasibility for veterinary applications; (2) long-term thermostability under typical farm cold-chain conditions, especially in high-temperature regions, has not been adequately evaluated; and (3) almost all data come from small-scale gnotobiotic pig studies, with no published field efficacy data against genetically diverse circulating strains. Furthermore, the potential for immune-mediated adverse events such as enhanced inflammation has not been systematically assessed in swine populations.

### 11.4 Epitope vaccine design

With the aid of bioinformatics and structural immunology methods, highly conserved T-cell and B-cell epitopes can be systematically screened from multiple proteins of Porcine Rotavirus Group A, thereby enabling the rational design of multi-epitope fusion antigens. Zhu et al. targeted the VP7 and VP8* proteins of Porcinovirus (PoRV). Using immunoinformatics tools, they predicted potential T-cell and B-cell epitopes. After screening based on multiple criteria, including antigenicity, non-toxicity, and immunogenicity, they linked eligible epitopes via linkers to construct a novel multi-epitope peptide vaccine construct. Computer simulations indicated that this vaccine protein can simultaneously induce both cellular and humoral immune responses (202). Similarly, de Oliveira Matos et al. combined the VP4, VP6, and VP7 proteins of rotavirus with the VP1 protein of norovirus to design the multivalent, multi-epitope vaccine ChRNV22 using immunoinformatics methods (203); Sharma et al. also targeted proteins such as VP4/VP7 and NSP2/NSP5, using immunoinformatics to screen for CD8^+^ T-cell, CD4^+^ T-cell, and B-cell epitopes, and then linked these epitopes with adjuvants and linkers to construct multi-epitope vaccine candidate molecules, all of which demonstrated good immunogenicity in computer simulations (204). The above studies indicate that the construction of “bead-like” multi-epitope vaccine candidates by serially combining highly conserved peptide segments derived from proteins such as VP6, VP7, VP4, and NSP4 has become a key strategy for designing a new generation of broad-spectrum porcine rotavirus vaccines. In porcine models, multi-epitope vaccines combined with appropriate adjuvants have been shown to induce cross-reactive T-cell and antibody responses against multiple homologous and heterologous rotavirus strains. Wen et al. administered a subunit vaccine consisting of a fusion of the P2 universal CD4^+^ T-cell epitope with a truncated VP8* protein to piglets via intramuscular injection. This induced serum virus-neutralizing antibodies and VP4-specific IgG prior to challenge; following challenge, it triggered intestinal and systemic IFN-*γ*-producing CD4^+^ T-cell responses, and the onset of diarrhea in the piglets was significantly delayed, and the duration of diarrhea and cumulative diarrhea scores were also significantly reduced (205). Tang et al. reported that after sows were immunized with a bivalent subunit vaccine (VP4*P[7] and VP4*p(23)), piglets acquired high levels of sIgA antibodies from colostrum. Following viral challenge, only a few piglets exhibited mild diarrhea or remained asymptomatic; intestinal tissue lesions were significantly reduced, and viral loads were lower than those in the control group (56). Furthermore, Parreno et al. combined the probiotic LGG as a mucosal adjuvant with the Lanzhou trivalent oral vaccine, which significantly enhanced both intestinal and systemic immune responses and improved protective efficacy against challenge with heterologous strains, further demonstrating that combining appropriate adjuvants can effectively enhance the cross-reactive immunity of multivalent/multi-epitope vaccines (132). Therefore, these studies collectively demonstrate that multivalent vaccines combined with appropriate adjuvants can induce cross-reactive immune responses against multiple homologous and heterologous rotavirus strains in porcine models and, to some extent, mitigate the severity of post-challenge diarrhea. Compared to whole-protein vaccines, epitope vaccines can precisely target the immune response to conserved regions, reducing the production of ineffective or even interfering antibodies against hypervariable regions. However, epitope vaccines have relatively weak immunogenicity, and optimizing epitope arrangement, delivery vectors, and adjuvant combinations remains key to improving their protective efficacy.

This precision-guided multi-epitope design strategy is a key component of the rational universal vaccine development framework shown in Figure 2, enabling focused immune targeting of conserved viral regions.

The primary limitation of current epitope vaccine research is that nearly all candidates remain at the in silico or small animal validation stage, with very few having undergone rigorous piglet challenge testing. Additional practical challenges include: (1) the weak intrinsic immunogenicity of short peptide epitopes, which requires pairing with potent adjuvants and optimized delivery systems; (2) the loss of conformational neutralizing epitopes, which are critical for high-titer antibody protection; and (3) the risk of rapid immune escape if single-point mutations occur within the targeted epitope regions.

### 11.5 Chimeric proteins and chimeric virus-like particles

Drawing on the strategy used with chimeric influenza hemagglutinin to direct the immune response toward conserved stem regions, the construction of a chimeric VP4 antigen based on the conserved stem region of rotavirus is an effective means of redirecting the immune response toward conserved functional domains. Komoto et al. successfully constructed a recombinant rotavirus expressing a chimeric VP4 using reverse genetics, providing direct technical validation for antigen redesign through VP4 molecular engineering (206). Building on this, a chimeric VP4 antigen was prepared by fusing the VP8 head from a rare P-type with the VP5 stem, which is relatively conserved across multiple P-types. By administering chimeric proteins with different VP8 heads but the same VP5 stem via sequential immunization, it is expected to progressively focus and amplify the cross-neutralizing antibody response targeting the conserved regions of VP5*. Drawing on the strategy of using chimeric influenza hemagglutinin to focus the immune response on the conserved stem region, constructing a chimeric VP4 antigen based on the conserved stem region of rotavirus is an effective means of redirecting the immune response toward conserved functional domains. By fusing VP8 heads from different P-types with the VP5 stem which is relatively conserved across multiple P-types to produce chimeric VP4 antigens, and by administering sequential immunizations with chimeric proteins carrying different VP8 heads but the same VP5 stem, it is expected that the cross-neutralizing antibody response targeting the conserved regions of VP5 can be progressively focused and amplified. Previous studies have validated at the molecular level the feasibility of using chimeric VP4 to present specific epitopes on the viral surface (206). Pijpers et al. found that neutralizing antibodies against VP5 can cross-react with viruses of various P genotypes (P1 to P8), and their immunoprotective efficacy against the virus is superior to that of VP8 (207). Nair et al. further confirmed in humans that antibodies targeting the VP5 region are key to mediating broad cross-protection (208). Drawing on the chimeric hemagglutinin (Cha) strategy used in influenza vaccines, Sunwoo et al. successfully directed the immune response toward the conserved stem region and induced cross-protection by implementing sequential immunization with different HA heads and a conserved stem in a swine model, demonstrating the feasibility of this “head-substitution, stem-conservation” design approach in pigs (208). Therefore, the strategy of chimeric VP4 antigen combined with sequential immunization holds promise for overcoming the limitations of conventional PoRV vaccines, which are constrained by the high variability of VP8*, and offers a new approach for achieving broad-spectrum immune protection against porcine rotavirus.

This chimeric antigen engineering strategy aligns with the “chimeric VP4” technical path of the universal vaccine framework in Figure 2, aiming to redirect immune responses from variable head regions to conserved stem regions.

While this chimeric antigen strategy is conceptually compelling, several critical gaps must be addressed. To date, chimeric VP4 approaches for porcine RVA have only been validated at the molecular construction level, with no published data on *in vivo* protective efficacy in pig challenge models. The immunodominance of variable VP8 head epitopes may still divert the immune response away from the conserved VP5 stem region, even with sequential immunization regimens. Furthermore, the manufacturing complexity and cost of chimeric proteins or VLPs may hinder large-scale production and commercial viability for veterinary use.

### 11.6 Overarching knowledge gaps and future research priorities

Across all universal vaccine strategies, four overarching scientific challenges currently limit progress in porcine RVA vaccine development:

#### 11.6.1 Lack of standardized cross-protection evaluation frameworks

There is no harmonized challenge protocol for assessing heterologous protection across different laboratories, including differences in challenge strains, inoculation doses, piglet ages, and efficacy endpoints. This makes direct comparison of vaccine candidates extremely difficult and hinders cumulative progress in the field.

#### 11.6.2 Incomplete definition of cross-protective immune correlates

While neutralizing antibodies are the accepted correlate for homotypic protection, the immune determinants of broad heterologous protection remain poorly characterized. Without validated quantitative correlates of cross-protection, rational vaccine and adjuvant design remains largely empirical.

#### 11.6.3 Maternal antibody interference in neonatal immunization

Nearly all novel vaccine candidates have been tested in gnotobiotic or antibody-free pig models. Their performance in the presence of maternally derived antibodies the standard scenario for commercial suckling piglets remains largely uncharacterized, representing a major translational gap.

#### 11.6.4 Field validation gap for novel platforms

Almost all next-generation vaccine and adjuvant strategies have only been evaluated in controlled experimental settings. Their efficacy, safety, and cost-effectiveness under commercial farm conditions with ubiquitous co-infections, variable herd immunity, and practical management constraints remain largely unproven.

Moving forward, the field should prioritize: (i) establishing international standardized cross-protection challenge protocols using well-characterized heterologous reference strains; (ii) defining quantitative immune correlates of broad cross-protection for both passive and active immunization; (iii) developing adjuvant and delivery strategies that can overcome maternal antibody interference; and (iv) conducting large-scale multicenter field trials to validate the real-world performance of next-generation vaccine candidates.

Collectively, these knowledge gaps represent the main bottlenecks restricting the translation of the universal vaccine strategies illustrated in Figure 2 from preclinical concepts to practical field applications.

## 12 Conclusion

Although the development and use of vaccines against porcine Group A rotavirus have spanned several decades, the virus remains one of the primary pathogens causing severe diarrhea and significant economic losses in newborn piglets across the global swine industry. This further underscores the urgent need for vaccines capable of providing broad, long-lasting, and cross-genotypic immune protection. The incorporation of adjuvants into vaccine formulations remains one of the most promising strategies for achieving this goal.

Traditional adjuvants (such as aluminum salts and oil emulsions), with their well-established safety profiles and ease of large-scale production, have laid a crucial foundation for existing commercial vaccines. However, their ability to induce broad-spectrum mucosal and cellular immune responses is relatively limited, driving the exploration of novel adjuvant strategies. Emerging adjuvants, represented by Toll-like receptor agonists, saponins, enterotoxin variants, and nanoparticles, have been shown to enhance cross-protective immunity against heterologous strains, enable antigen dose savings, and broaden the spectrum of immune recognition by targeting specific innate immune pathways and mucosal presentation routes. Notably, most current evidence remains at the level of enhanced immunogenicity or partial homologous protection, and the ability of adjuvants to fully overcome genotype mismatch and confer broad heterologous protection requires further rigorous validation.

Concurrently, novel antigen delivery systems based on liposomes, polymeric nanoparticles, and plant-derived nanoparticles are being employed to improve antigen stability and mucosal delivery efficiency. The development of mucosal immunization routes, such as oral and intranasal administration, aims to more effectively activate local immune responses at the intestinal mucosa the primary entry point for viral invasion. Furthermore, surface modification of traditional adjuvants, formulation optimization, and the combined use of multiple immunostimulatory molecules provide clinically feasible technical pathways for enhancing the performance of existing vaccines.

Looking ahead, rational design of next-generation adjuvants and delivery systems, guided by deeper understanding of immunological mechanisms, will be central to developing a porcine Group A rotavirus vaccine that combines high immunogenicity, good safety, scalability, and broad, effective protection against multiple G/P genotype strains. Addressing core knowledge gaps including standardized cross-protection evaluation, defined cross-protective correlates, maternal antibody interference, and field validation will be critical to translating promising preclinical strategies into real-world impact. This review seeks to provide a systematic summary of these efforts.
